# Active surveillance of papillary thyroid carcinoma in Latin America: a scoping review

**DOI:** 10.20945/2359-4292-2023-0495

**Published:** 2024-09-24

**Authors:** Pedro Garnier Manfio¹, Lucas Albuquerque Chinelatto², Flávio Carneiro Hojaij³

**Affiliations:** 1 Universidade de São Paulo Faculdade de Medicina São Paulo SP Brasil Faculdade de Medicina, Universidade de São Paulo, São Paulo, SP, Brasil; 2 Universidade de São Paulo Faculdade de Medicina Hospital das Clínicas São Paulo SP Brasil Residência em Otorrinolaringologia, Hospital das Clínicas, Faculdade de Medicina, Universidade de São Paulo, São Paulo, SP, Brasil; 3 Universidade de São Paulo Faculdade de Medicina LIM 02 – Departamento de Cirurgia São Paulo SP Brasil LIM 02 – Departamento de Cirurgia, Faculdade de Medicina, Universidade de São Paulo, São Paulo, SP, Brasil

**Keywords:** Thyroid cancer, active surveillance, Latin America, papillary carcinoma

## Abstract

Thyroid nodules are a very common finding and have a malignancy rate of 7%-15%. Some malignant nodules have an indolent behavior and may not affect mortality if left untreated. Active surveillance (AS) is a strategy to prevent overtreatment in patients with papillary thyroid microcarcinomas (PTMCs). This review was conducted to evaluate the status of AS for low-risk PTMC in Latin America, including cultural and logistical challenges, disease progression data, and financial viability. We searched PubMed (MEDLINE), SciELO, LILACS, and Web of Science for articles published after 2014 and enrolling adult Latin American patients. Articles cited in the selected studies were also retrieved. We analyzed the AS protocols, technical or logistical challenges, patient adherence, reasons for AS interruption, surgical conversion rates, duration of AS, and disease progression during AS in our region. Three articles were included in the analysis, all of which considered AS a viable option and reported tumor progression and outcomes similar to those reported in other countries. Neck ultrasound and serum levels of thyroglobulin, thyroid-stimulating hormone (TSH), thyroxine (T4), and antithyroglobulin antibodies were included in the follow-up. No cases of new distant metastases were reported, and the outcomes were favorable when surgery was required. Anxiety was the main reason for AS interruption. We conclude that AS can be an acceptable approach and is safe and effective in Latin America, although more prospective studies are needed to consolidate this strategy in our region. Adequate infrastructure, follow-up, and patient education, as well as multidisciplinary healthcare teams trained in conducting AS must be ensured for successful results.

## INTRODUCTION

Thyroid nodules are a common finding, with an estimated prevalence of 19%-68% in adults when detected using high-resolution ultrasound (1,2). However, only 7%-15% of these nodules are malignant (3). Fine-needle aspiration (FNA) is the preferred method for distinguishing between malignant and benign nodules. The most prevalent benign nodules are colloid nodules, macrofollicular adenomas, and lymphocytic thyroiditis. Papillary thyroid cancer (PTC) is the most common malignant histologic finding, representing 90% of all differentiated thyroid cancers (3).

Even when considering the risk of malignancy, FNA is not advised for nodules smaller than 1 cm, according to the 2015 American Thyroid Association (ATA) guidelines (3). This recommendation is endorsed by the American College of Radiology Thyroid Imaging, Reporting and Data System (ACR TI-RADS) (4) and the European Thyroid Association TIRADS (EU-TIRADS) (5) due to the indolent behavior of small size (≤1 cm) PTCs, also known as papillary thyroid microcarcinomas (PTMCs). In the last decades, easier access to neck ultrasound and FNA has significantly increased the prevalence of PTMCs with little effect on morbidity and mortality and very low tumor progression probability (6,7). A PTMC without extrathyroidal extension, lymph node metastasis, distant metastasis, or unfavorable cytology is classified as a low-risk PTMC. Patients with low-risk PTMC often undergo surgery and medical treatment without clear benefits (8); for these patients, AS is an alternative to surgery.

The use of AS in low-risk PTMC began in the 1990s in Japan as a way of preventing overtreatment of these tumors (9). This approach comprises periodical clinical and ultrasonographic examination to assess eventual disease progression, in which case surgical intervention is indicated. Additionally, AS is frequently used in patients with high surgical risk, in those expected to have a short remaining life span, or in individuals with concurrent health issues requiring intervention prior to thyroid surgery. For patients with low-risk PTMC, long-term AS is a viable alternative to avoid surgery, which is associated with costs along with potential complications and the need for lifelong hormone replacement therapy (3).

In Japan, where AS is a well-established practice (7), FNA is recommended in nodules ≥ 5 mm for the detection of PTMC, and AS is offered as a treatment option in this situation (10). When low-risk PTMC is confirmed, diagnostic communication and explanations about its good prognosis without surgical treatment are key to reducing the patient's anxiety and avoiding unnecessary surgery.

During the COVID-19 pandemic, many oncologic surgeries were strategically delayed. In this context, AS protocols became more common worldwide as an option to guarantee continued care in patients for whom surgery was delayed.

In Latin America, AS is rarely done (11). There is great anxiety among patients and physicians regarding the diagnosis of cancer, logistical difficulties in performing periodic neck ultrasound exams for a growing number of patients with low-risk PTMC, and only a few studies assessing AS outcomes.

The present review was conducted to evaluate the status of AS protocols for low-risk PTMC in Latin America with regard to cultural and logistical challenges, disease progression data in the Latin American population, and financial viability compared with surgery. The primary aim of the study was to evaluate the acceptance of AS for low-risk PTMC in Latin America. Secondary study aims were to evaluate the protocols and parameters used for AS, the safety of this strategy, and its financial viability compared with surgery in Latin America.

## METHODS

This study is a scoping review of AS strategies in adult Latin American patients diagnosed with low-risk PTMC. The articles were selected after a systematic search strategy in the following databases: PubMed (MEDLINE), SciELO, LILACS, and Web of Science. The search strategy included the following terms: ("Papillary Thyroid Cancer" OR "Papillary Thyroid Carcinoma" OR "Micropapillary Thyroid Cancer" OR "Micropapillary Thyroid Carcinoma") AND (Screening OR Screen* OR "Active Surveillance" OR "Surveillance") AND ("South America" OR "Latin America" OR Brazil OR Argentina OR Colombia OR Chile OR Bolivia OR "Costa Rica" OR Cuba OR "Dominican Republic" OR Ecuador OR "El Salvador" OR Guatemala OR Haiti OR Honduras OR Mexico OR Nicaragua OR Panama OR Paraguay OR Peru OR "Puerto Rico" OR Uruguay OR Venezuela).

The inclusion criteria were articles written in English, Portuguese, Spanish, or French, published after 2014, and focused on AS for thyroid cancer. This timeframe was chosen to ensure the inclusion of articles published after the 2015 ATA guidelines. The articles cited by the selected studies that met all the inclusion criteria but were not initially included were also reviewed. Articles were excluded if the median patient age was below 18 years or if the study included a non-Latin American population. Articles that mentioned AS but did not focus on its application in Latin America or the outcomes of this strategy were also excluded.

Duplicate articles were initially excluded using the reference manager Paperpile. After excluding duplicates, at least one author read the title and abstract of the studies to select those for full-text review. Two authors fully read the selected articles. In the case of studies with repetitive data, the most recent study was included.

The selected studies were analyzed for quantitative and qualitative data, AS protocols, technical or logistical challenges, patient adherence, reasons for AS interruption, surgical conversion rates, AS duration, and disease progression during AS. We calculated the surgical indication rates of each study by dividing the total number of patients undergoing AS who received a surgical indication due to disease progression by the total number of patients undergoing AS. We then divided the obtained result by the median AS duration (in years). The final results were multiplied by 100 to facilitate comparison. Quantitative data were described in simple parametric factors using Google Sheets.

To evaluate the quality of the selected studies, we applied the Newcastle-Ottawa Scale. Two authors independently evaluated each study and graded it accordingly. When the evaluators had different opinions on a topic, they debated until reaching a consensus. The studies were deemed of low, moderate, or high quality according to the number of stars (points) they received: 0-3 low, 4-6 moderate, and > 7 high.

## RESULTS

**Figure 1:**
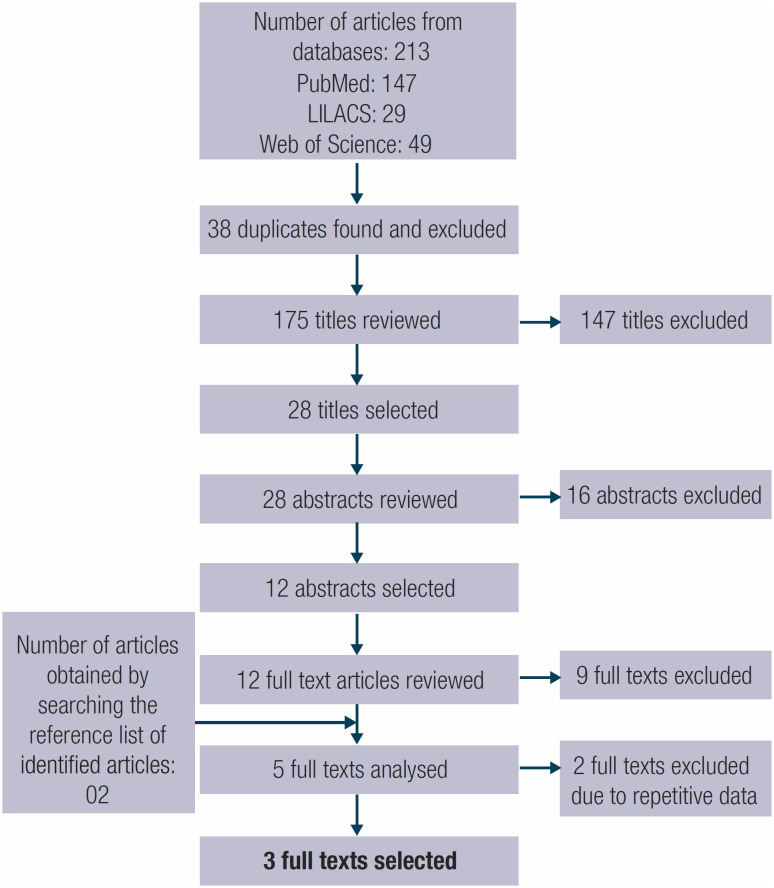
Preferred Reporting Items for Systematic Reviews and Meta- Analyses (PRISMA) diagram illustrating the article inclusion flowchart.

The initial search retrieved 213 results. After excluding duplicates using Paperpile, 175 unique titles were analyzed. Titles not referring to PTC treatment options were excluded, and 28 articles remained for abstract analysis. After excluding papers not addressing AS in their abstracts, the remaining articles were read in full. During full-text review, studies were excluded for the following reasons: without Latin American patients, without data specifically about AS, and reviews of other studies. Finally, by analyzing the reference list of the selected articles, two additional studies were identified and added to the review. The initial review included five articles comprising two studies from Argentina, one from Brazil, and two from Colombia, all of which enrolled patients from the local population of each respective country. Two studies were conducted by the same Argentinian center (12,13) and two by the same Colombian center (14,15). To avoid overlapping data, we analyzed the most recent study from each group (12,15), both of which included a larger population than the previous study from the same center (Figure 1).

The three articles selected comprised cohort studies. Based on the Newcastle-Ottawa Scale, one study was classified as having moderate quality (15), and the other two studies as having low quality (12,16). The Colombian study (15) received 5 out of 9 stars, while both the Brazilian and Argentinian studies (12,16) received 3 out of 9 stars.

All selected studies considered AS for low-risk PTMC as a viable option in Latin America and had positive results for this treatment approach (Table 1). None of the three studies reported any deaths attributable to low-risk PTMC. Surgeries after a period of AS had excellent outcomes (12), and no cases of new distant metastases were reported by the selected articles (12,15,16). Two cases of lymph node metastases were reported in the Argentinian study (4.8%) (12). None of the selected studies reported data on cost comparison between surgery and AS.

**Table 1:** Active surveillance protocols, number of patients included who underwent active surveillance, disease progression, and surgery after active surveillance

Author, year	Country	Protocol	N	AS acceptance, n (%)	Median age of patients in AS, years (range)	Sex distribution of patients in AS, n (%)	Median surveillance duration, months (range)	Disease progression	Surgery during AS indicated	
									Disease progression*, n (%)	Non-disease related, n (reason)
Smulever and cols., 2020	Argentina	Neck US and Tg, TSH, and TgAb levels semiannually for 1 year, then annually	164	41 (25%)	41.5 (15-79)	Female, 36 (88%); male, 5 (12%)		Tumor growth > 3 mm, 6 (14.6%); Tg doubling in < 1 year, 1 (2.4%); lymph node metastases, 2 (4.8%)	4 (9.75%)	8 (anxiety)
Rosario and cols., 2019	Brazil	Neck US and TSH and T4 levels every 6 months	95	77 (80%)	52 (23-81)	Female, 61 (79%); Male, 16 (21%)	(#)	Tumor growth associated with a suspicion of extrathyroidal invasion, but no lymph node metastases 1.3% (1)	1 (1.3%)	2 (#)
Sanabria, 2020	Colombia	Neck US semiannually for 1 year, then annually plus consultation if red flags**	102	Only patients who accepted the strategy were reported	50 (22-86)	Female, 85 (83%); Male, 17 (17%)	13.9 (0.2-112)	Tumor growth > 3 mm, 11 (10.8%); growth > 30% in diameter, 15 (14.7%); growth > 50% in volume, 26 (25.5%)	8 (7.84%)	5 (#)

* Surgery was not indicated in all cases of disease progression.

** If clinical symptoms or lymph node enlargement. # Not reported. Abbreviations: AS, active surveillance; T4, thyroxine; TSH, thyroid-stimulating hormone; US, ultrasound.

In all studies, AS included periodic neck ultrasound. One study monitored Tg and TSH levels (12), another monitored T4 levels (16), and a third study monitored TgAbs (12). One protocol provided medical consultations if any symptoms emerged (15).

The rates of surgical indication per 100 patients were 3.12 (12) and 6.77 (15). The surgical indication rate could not be calculated in one of the studies (16), as it did not report the median AS duration.

All three studies also provided considerations regarding the implementation of AS in the context of the Latin American healthcare system. The challenges pointed out by the studies included anxiety of the patients (12); mindset of interventionists, patients, and physicians regarding treatment (12); preference of physicians for surgery or not transmitting safety regarding AS (16); lack of knowledge about indications and results of AS by healthcare personnel (15); lack of comprehensive care in the diagnosis and management of patients (15); intolerance to uncertainty by some members of the medical team (15); fear of lawsuits (15); and position of scientific societies against the strategy (15).

## DISCUSSION

Our main finding in the present review was that AS can be accepted by patients in Latin America if sufficient explanations and patient education are offered. The analyzed studies indicated AS to be safe in Latin America, with data revealing tumor progression and outcomes similar to those obtained in other parts of the world (9,13,15,17). In all three studies included in the present review, no deaths due to low-risk PTMC were recorded (12-16). Surgeries after a period of AS had excellent outcomes and no cases of new distant metastases were reported in the selected articles.

The three studies analyzed had similar outcomes, although no standardized AS protocol is available for low-risk PTMC in our region: the follow-up protocols of all three studies included neck ultrasound, but only two of them also included TSH, and one included both Tg and TgAb. One study monitored T4 levels. One study recommended semiannual follow-ups, while two of them recommended follow-ups every 6 months during the first year and annually thereafter. The reports about the percentages of tumor growth > 3 mm of 10.8% (15) and 14.6% (12) are aligned with data described in other countries. For example, an American study (17) found that only 10%-15% of low-risk PTCs (intrathyroidal tumors ≤ 1.5 cm) will grow ≥ 3 mm in the first 5 years of AS. The same study also suggested that low-risk PTCs between 1.0 and 1.5 cm have a low likelihood of growth, similar to the findings for tumors smaller than 1 cm.

The percentage of patients for whom surgery was recommended due to disease progression was higher in the group that presented a longer median surveillance time, suggesting that longer AS duration results in more surgical indications. However, a Japanese study (18) showed that patients followed up for 20 years, compared with those followed up for 10 years, did not show a substantially different percentage of tumor enlargement > 3 mm (6.6% versus 4.7%, respectively) or novel lymph node metastasis (1.6% versus 1%), suggesting that low-risk PTMCs that remain stable in the first years of AS have a smaller chance of progression than recently discovered ones.

Surgical indication rates per year were 3.12% (12) and 6.77% (15). Comparing these data with those from Kuma Hospital in Japan (18) – one of the pioneer centers of AS for low-risk PTMC, with a surgical indication rate of 1.68% per year – surgery is indicated slightly more frequently in Latin America. Nevertheless, it is important to point out that some situations considered disease progression in the analyzed Latin American studies are not considered necessarily surgical indications by the Consensus Statements from the Japan Association of Endocrine Surgery (JAES) Task Force on Management for Papillary Thyroid Microcarcinoma (19). According to the JAES Consensus statements, the indications for surgery after AS for Papillary Thyroid Microcarcinoma, apart from patient preference, include tumor diameter ≥ 13 mm and the emergence of new lymph node metastasis, other thyroid disease, or parathyroid disease requiring surgery.

Cancer-related anxiety is a clear challenge for AS implementation in Latin America. In the Argentinian trial (12,13), patients chose surgical treatment in 75% of the cases due to concern about disease progression. However, the Brazilian study (16) reported an 80% acceptance rate for AS, mainly because physicians expressed their preference for AS when questioned by the patients. In two-thirds of the cases reported in the Brazilian study, the patients wanted to know the doctors’ preferences before deciding on AS or surgery. This demonstrates that doctors and other healthcare workers who are willing to educate patients about the advantages of AS are crucial for the acceptance of this protocol. Moreover, between all physicians caring for the patient and the healthcare team, there must be a consensus regarding the management of low-risk PTMC to avoid dissonant information and patient anxiety, as suggested by the Brazilian Society's consensus on AS for thyroid cancer (20).

As pointed out by Rosario and cols. (16), we also believe that the Internet may facilitate AS acceptance by patients. Knowledge diffusion about the possibilities of AS allows patients to make a more educated decision. On the other hand, the population's educational level is a possible barrier (14), considering that Latin America is a developing region with significant education challenges (21). This affects decision-making in the treatment of low-risk PTMC because it hinders the patient's ability to ponder the pros and cons of surgery and AS.

The lack of specifically trained multidisciplinary healthcare teams for AS protocols in Latin America is an impediment, but recent studies and position statements, such as the one by the Brazilian Society of Endocrinology and Metabolism (SBEM) (20) defending that AS is an appropriate initial choice in selected patients, might favor the implementation of the strategy in Latin America in the next years.

Another aspect to be contemplated is financial viability. No data about cost comparisons between surgery and AS were found in the three selected studies. Even so, we consider that AS can reduce the total costs related to low-risk PTMC. It can reduce surgical indications by using simple blood tests and neck ultrasound evaluations, avoiding the many surgery-related expenses, such as those related to hospitalizations and materials. This point of view is reinforced by an Argentinian article (22), which concluded that costs associated with AS could be three to four times lower than those with thyroid surgery. This cost-effectiveness would favor AS, but more studies must be conducted to compare the costs of surgery and AS in other Latin American countries.

One must still take into consideration that all the AS protocols that we analyzed recommended neck ultrasound to evaluate the nodules. Availability of good ultrasound equipment and experienced neck ultrasonographers are necessary for AS, considering that this is an inherently operator-dependent imaging modality and millimetric tumor growth may not be noticed if the equipment is not optimal or the technician is not experienced. For patients living in rural regions or locations far from medical centers, periodic consultations, blood tests, and ultrasound evaluations require travel to urban areas, which often have poor transportation infrastructure, influencing their decision to opt for surgical treatment. Considering this, the healthcare system must facilitate access with flexibility to schedule appointments for consultations or exams. We acknowledge that patients who miss follow-up appointments have a chance of disease progression and could be cured of low-risk PTMC with surgery, and for this reason, AS should not be indicated if follow-up is unfeasible in particular cases.

The main limitation of this study is that it analyzed only three articles. Due to the absence of standardized AS protocols in Latin America, data comparison is not ideal. Some differences in outcomes, such as surgical conversion rates and disease progression, are not entirely explainable with the available data. We point out, as possible factors for those discrepancies, the different populations and variations in ultrasonographic measurements due to different equipment and operators.

Although the information collected favors the implementation of AS in Latin America, more prospective studies are needed to consolidate the safety, effectiveness, and viability of the strategy in this part of the world. There is a lack of meta-analyses about the topic in Latin America due to the scarcity and limitations of the observational studies. We believe that a standardized AS protocol for Latin America would be advantageous for improving the quality of data on the topic.

In conclusion, the data analyzed in the present review favors AS protocols for low-risk PTMC in Latin America. The three studies conducted by groups in Argentina, Brazil, and Colombia had outcomes similar to those in other parts of the world, suggesting that AS is safe and effective in Latin America. Still, more prospective studies are needed to consolidate this strategy in our continent. Adequate infrastructure, follow-up, and patient education, along with multidisciplinary healthcare teams specifically trained to conduct this kind of protocol, must be ensured for successful outcomes.
